# An overview of natural herbal extracts and compounds for combating porcine epidemic diarrhea virus

**DOI:** 10.3389/fvets.2025.1557198

**Published:** 2025-06-06

**Authors:** Liyan Wang, Zongyi Bo, Liumei Sun

**Affiliations:** ^1^Jiangsu Key Laboratory of Sericultural and Animal Biotechnology, School of Biotechnology, Jiangsu University of Science and Technology, Zhenjiang, China; ^2^Key Laboratory of Silkworm and Mulberry Genetic Improvement, Ministry of Agriculture and Rural Affairs, Sericultural Scientific Research Center, Chinese Academy of Agricultural Sciences, Zhenjiang, China; ^3^Joint International Research Laboratory of Agriculture and Agri-Product Safety, The Ministry of Education of China, Yangzhou University, Yangzhou, China; ^4^Jiangsu Co-Innovation Center for the Prevention and Control of Important Animal Infectious Disease and Zoonoses, College of Veterinary Medicine, Yangzhou University, Yangzhou, China

**Keywords:** porcine epidemic diarrhea virus, natural herbal products, antiviral, single-herb extracts, compound herbal formulations

## Abstract

Porcine epidemic diarrhea virus (PEDV) is the etiological agent responsible for the acute infectious intestinal disease known as porcine epidemic diarrhea (PED), which results in severe diarrhea in piglets and causes substantial economic losses to the global swine industry. Currently, no specific therapeutic agent is available for the clinical treatment of PEDV, highlighting the urgent need to screen antiviral compounds and investigate antiviral mechanisms to develop effective antiviral therapies. Using active natural ingredients in animal husbandry represents a significant contemporary trend. Natural herbal products offer numerous advantages, including abundant availability, diverse biological activities, and low toxicity and side effects, as well as a reduced likelihood of developing drug resistance. These attributes position them as valuable resources for the development of effective anti-PEDV drugs. This paper provides a comprehensive review of the current research on the inhibitory effects of herbal monomers (isolated compounds), single-herb extracts, and compound herbal formulations (derived from research) against PEDV. The aim is to establish a theoretical foundation for the screening and development of therapeutic and prophylactic agents targeting PEDV.

## 1 Introduction

Porcine epidemic diarrhea (PED) is a highly transmissible enteric infectious disease in swine, clinically characterized by acute enteritis, vomiting, watery diarrhea, and dehydration, and is caused by the porcine epidemic diarrhea virus (PEDV) (1). The disease can result in up to 100% mortality among lactating piglets (2). Initially identified in the United Kingdom in the early 1970s (3), PED was subsequently reported in China in the early 1980s. Various variant of the virus became widespread in 2010, leading to significant economic losses within the Chinese pig industry (4, 5). This situation poses a substantial threat to global food safety and has a profound impact on agricultural practices and economies worldwide (6). Currently, no specific pharmacological treatment for PEDV is available in clinical settings. Consequently, it is crucial to continue the screening of antiviral compounds, investigate the pathogenesis of antiviral mechanisms, and develop highly effective antiviral agents.

In recent years, a series of advances have been made in research against PEDV. In terms of vaccine development, traditional inactivated and weakly virulent vaccines have been widely used in practice, but there are limitations in their protective effects. With the continuous development of genetic engineering technology, new types of vaccines (e.g., DNA vaccines, mRNA vaccines, and virus-like particles vaccines) have gradually emerged and demonstrated higher immunogenicity and protective efficacy (7, 8). However, the existing PEDV vaccines are often unable to provide ideal immunoprotection in the face of emerging mutant strains. Small-molecule drugs such as the protease inhibitor GC376 and the anticancer drug RAF265 have also demonstrated efficacy in inhibiting PEDV replication (9, 10).

However, the application of veterinary antiviral drugs in food-producing animals may lead to the presence of drug residues in animal-derived products such as meat and milk. Assessing the risk associated with the residual toxicity of antiviral drugs to consumer health presents significant challenges. Consequently, the pursuit of more efficient and safer pharmaceuticals is paramount to ensuring consumer food safety. Empirical studies have demonstrated that natural herbal products, including phytotherapeutic agents, botanicals, and other natural compounds or synthetic derivatives, serve as a vital resource for the development of novel antiviral agents (11–13). They are distinguished not only by their multi-component, multi-pathway, and multi-target mechanisms of action but also by their abundant sources, diverse biological activities, symptomatic treatment capabilities, and a reduced propensity for developing drug resistance (14). These characteristics confer advantages in safety, efficacy, and cost-effectiveness, positioning them as promising candidates for the development of potent anti-PEDV drugs. This paper reviews recent studies on the inhibition of PEDV by various herbal monomers, single-flavor herbal extracts, and compound herbal preparations, aiming to provide a theoretical foundation for the screening and study of drugs for the treatment and prevention of PEDV.

## 2 Progress on the anti-PEDV effects of herbal monomer substances

**Figure 1:**
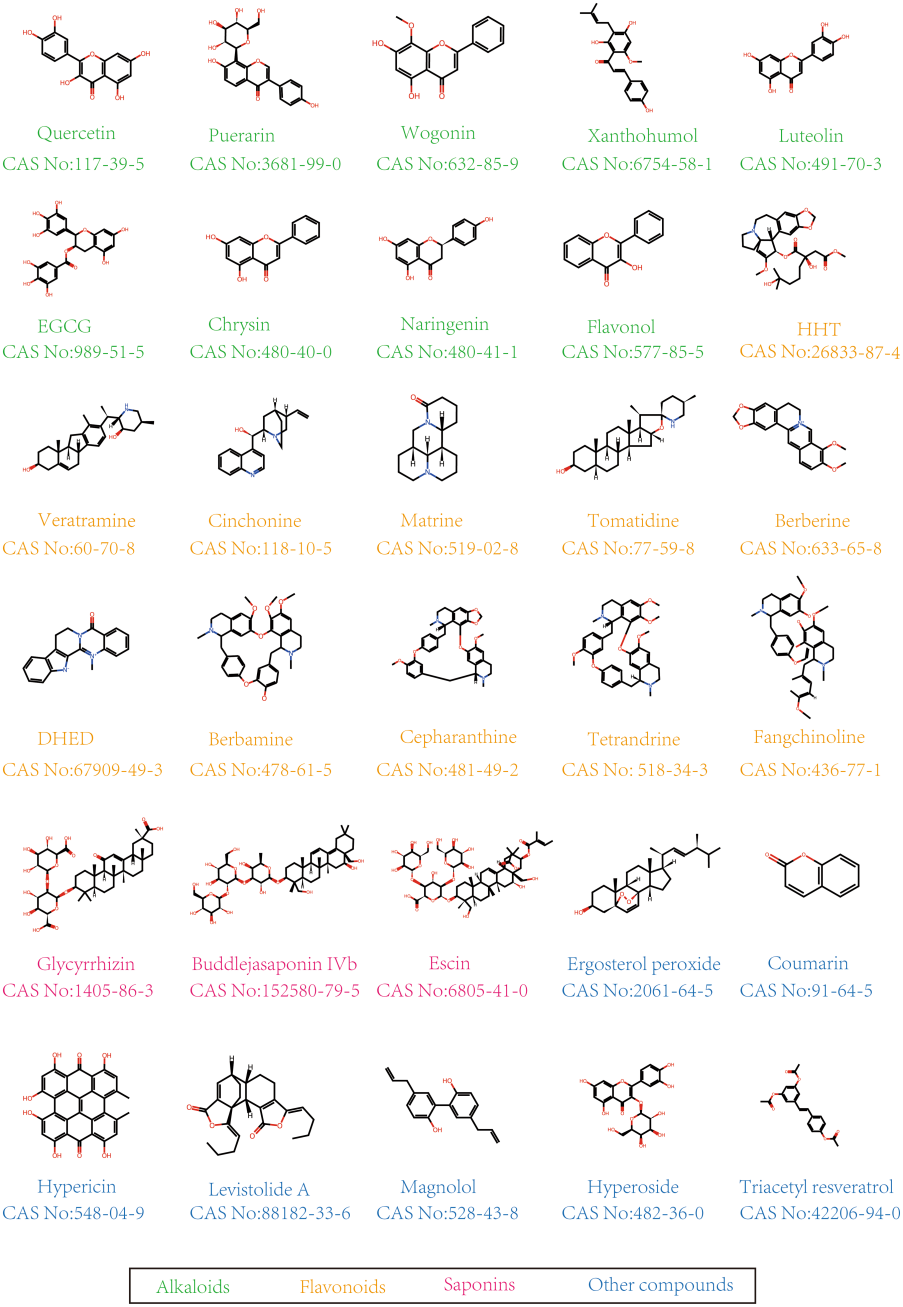
Chemical structure of anti-PEDV compounds.

In this study, herbal monomers are defined as single chemical compounds isolated and purified from herbs, which possess a definite molecular structure and biological activity. Currently, plant monomers with anti-PEDV activity can be classified into flavonoids, alkaloids, polysaccharides, saponins, and others. The chemical structures of these natural compounds with anti-PEDV activity are presented in Figure 1.

### 2.1 Research on the anti-PEDV activity of flavonoids

Flavonoids represent a category of polyphenolic secondary metabolites prevalent in a diverse range of fruits, vegetables, herbs, stems, and cereals. Based on their molecular architecture, flavonoids are further categorized into subgroups such as flavonoids, flavonols, chalcones, isoflavonoids, anthocyanins, and biflavonoids, among others. These compounds are known to modulate cellular immune functions and influence key cellular enzyme activities. Additionally, they exhibit antioxidant, anti-inflammatory, antibacterial, and anticancer properties. Consequently, flavonoids have been extensively utilized in the domains of nutraceuticals, pharmaceuticals, medicine, and cosmetics (15).

Research has demonstrated that various flavonoids exhibit significant inhibitory effects on the PEDV. Notably, compounds such as puerarin (PR), wogonin, xanthohumol, luteolin, epigallocatechin-3-gallate (EGCG), chrysin, naringenin, quercetin and flavonol, all classified as flavonoids, have been identified as effective agents in restricting PEDV. Among these, PR, an isoflavone compound derived from *Pueraria lobata*, is recognized as a traditional Chinese herbal medicine (16). Wogonin, another flavonoid, is extracted from the roots of *Scutellaria baicalensis*, a member of the Lamiaceae family, and is known for its extensive biological activities (17). Xanthohumol is a flavonoid derived from *Humulus lupulus* L., known for its antioxidant properties and specific antiviral activity (18). Luteolin is a common bioflavonoid found in various fruits and vegetables, and has a variety of beneficial medicinal properties, such as anti-tumor, anti-inflammatory, cardioprotective and neuroprotective effects (19). EGCG, a predominant flavonoid component of *Camellia sinensis* (L.) polyphenols, exhibits significant antiviral activity against a range of viruses, including the human immunodeficiency virus, influenza virus, encephalitis virus, and porcine reproductive and respiratory syndrome virus. Chrysin, the principal active compound extracted from *Wisteria sinensis* of the Leguminosae family and *Pinus koraiensis* of the Pinaceae family, also demonstrates noteworthy biological activity. Naringenin is a secondary metabolite found in *Citrus sinensis* belonging to the Rutaceae family. Quercetin, a natural polyhydroxy flavonoid, is derived from rutin. Flavonol, a natural flavonoid compound, is extracted from various sources including herbs, vegetables, citrus fruits, and tea. Flavonol exhibits numerous pharmacological properties, such as anti-inflammatory, antioxidant, and antibacterial activities, among others (20, 21).

The study revealed that the majority of flavonoids have the capacity to bind to the cysteine protease (Mpro or 3CLpro) protein of PEDV, thereby inhibiting viral replication. Specifically, wogonin modulates PEDV replication by targeting the substrate binding site of the PEDV 3CLpro, effectively inhibiting 3CLpro activity. This action significantly impedes the entry, replication, and release phases of PEDV, and also possesses the ability to directly inactivate PEDV *in vitro* (22). Additionally, xanthohumol has been shown to inhibit the activity of the coronavirus 3CLpro, thereby suppressing the replication of both SARS-CoV-2 and PEDV in cellular environments, and acts as a potent pan-inhibitor against a variety of coronaviruses (23). Luteolin has been shown to inhibit the activity of PEDV 3CLpro, the SI values on Vero and IPEC-J2 cells were 21.44 μM and 3.48 μM, as well as the internalization, replication, and release of PEDV (24). Notably, luteolin inhibits PEDV internalization independently of PEDV-S binding to pACE2. Notably, the susceptibility of PEDV to luteolin diminished following ten passages in cell culture, which coincided with increasing concentrations of luteolin. Chrysin and naringenin may exert antiviral effects by interacting with viral proteases, specifically 3CLpro or PLP2, thereby disrupting their role in the synthesis of PEDV non-structural proteins or interfering with viral replication (25). Quercetin has been demonstrated to inhibit PEDV replication by interacting with the three active sites of 3CLpro: Cys144, Asn141, and His162 (26). PEDV infection facilitates the accumulation of lipid droplets, while quercetin inhibits viral replication by reducing lipid droplet accumulation through the downregulation of NF-kB signaling and the levels of IL-1β, IL-8, and IL-6 (27). Additionally, flavonol has been found to inhibit PEDV replication in Vero and IPEC-J2 cells, the SI were 20.37 μM and 33.78 μM, and it is predicted to interact with PEDV 3CLpro through hydrogen bonds and hydrophobic forces (28). EGCG interacts with the 3CLpro and PLPro proteases of SARS-CoV-2, thereby impeding viral replication and eliciting an antiviral response within the host organism. Previous research has demonstrated that EGCG can inhibit PEDV infection by obstructing viral adsorption, entry, replication, and assembly (29). However, it remains to be determined whether EGCG also binds to the 3CLpro and PLPro proteases of PEDV. Recent studies have identified that baicalein and baicalin can similarly bind to the active site and binding pocket of PEDV 3CLpro, thereby inhibiting early viral replication during the post-entry stage of the PEDV life cycle, with IC50 values of 9.50 ± 1.02 μM and 65.80 ± 6.57 μM, respectively, (30).

Furthermore, flavonoids have been demonstrated to inhibit PEDV replication through modulation of the NF-κB signaling pathway and alteration of host immune-inflammatory factor expression. Experimental studies, both *in vivo* and *in vitro*, have revealed that PR effectively inhibits the activation of the NF-κB signaling pathway in PEDV-infected Vero cells and piglets. This inhibition leads to a reduction in PEDV-induced inflammatory cytokine levels, attenuation of intestinal damage, and regulation of gut microbiota in piglets. Consequently, PR mitigates the decline in growth performance in piglets and exhibits both antiviral and anti-inflammatory properties (31).

### 2.2 Research on the anti-PEDV activity of alkaloids

Alkaloids, a natural class of nitrogenous alkaline compounds found in plant secondary metabolites, are widely distributed. The extraction of alkaloids is relatively straightforward, time-efficient in practical applications, yields superior extraction results, generates minimal pollution (32). Research has demonstrated that various alkaloids, including homoharringtonine (HHT), veratramine (VAM), cinchonine, matrine, tomatidine, berberine, dehydroevodiamine (DHED), berbamine, several bis-benzylisoquinoline alkaloids and carbazole alkaloids, exhibit anti-PEDV properties.

HHT, a plant-derived alkaloid, has been shown to effectively inhibit the replication of multiple viruses, such as vesicular stomatitis virus (VSV), Newcastle disease virus (NDV), PEDV, herpes simplex virus type 1 (HSV-1), and pseudorabies virus (PRV), thereby demonstrating a broad-spectrum antiviral effect (33). Furthermore, a concentration of 300 nM of HHT alone is sufficient to achieve a significant antiviral effect. When combined with hydroxychloroquine (HCQ), the required concentration of HHT is reduced to 150 nM to achieve a comparable anti-PEDV effect (34). VAM, a piperidine alkaloid, has been shown to inhibit PEDV replication by suppressing PEDV-induced phosphatidylinositol 3-kinase/protein kinase B (PI3K/Akt) signaling pathways and interfering with the cellular mechanisms essential for PEDV entry into host cells. Consequently, VAM represents a promising lead compound for the development of anti-PEDV therapeutics (35). In contrast, cinchonine, a quinoline-type alkaloid, primarily exerts its antiviral effects during the early stages of PEDV infection, specifically during the adsorption and invasion phases. It facilitates autophagy in Vero CCL81, ST, and LLC-PK1 cell lines, thereby inhibiting PEDV infection (36). Matrine, a tetracyclic quinolizidine alkaloid, impedes PEDV adsorption and entry by targeting S proteins and suppresses PEDV replication by inducing apoptosis via the MAPK signaling pathway (37). Furthermore, the steroidal alkaloid tomatidine markedly inhibits PEDV replication by directly obstructing 3CLpro activity, the SI values on Vero cells were 13.25 μM (38). DHED, a quinazolinocarboline alkaloid, is derived from traditional Chinese medicine *Tetradium ruticarpum* (Wu-Zhu-Yu). DHED was demonstrated to inhibit the entry, replication, and assembly stages of the PEDV life cycle. The mechanism underlying DHED’s anti-PEDV activity involves the regulation of the MAPK signaling pathway through the suppression of phosphorylated ERK1/2 activation in Vero CCL81 cells (39). The bisbenzylisoquinoline alkaloids Cepharanthine (CEP), tetrandrine (TET), and fangchinoline (FAN) have been identified as compounds capable of obstructing all stages of the viral cycle. Specifically, FAN reduces viral activity by either interfering with viral adsorption and entry or by directly diminishing viral activity (40). FAN attenuates the activity of Cathepsin L and Cathepsin B by inhibiting lysosomal acidification, thereby preventing the entry of PEDV into host cells. This inhibition is further supported by a reduction in autophagy within IPEC-J2 cells (41). Berbamine, a natural compound derived from the traditional Chinese medicinal plant *Phellodendron amurense* Rupr., is classified under Bis-Benzylisoquinoline Alkaloids and is known for its anti-tumor, immunomodulatory, and cardiovascular properties. It has been reported to impede PEDV proliferation both *in vivo* and *in vitro*, primarily in the replication phase of the PEDV life cycle in Vero cells. Furthermore, viral non-structural proteins 3 and 16 (Nsp3 and Nsp16) have been predicted to interact with berbamine based on autodock simulations. Notably, berbamine has demonstrated efficacy in mitigating intestinal damage and inflammatory responses in piglets infected with PEDV (42).

Evidence suggests that, in addition to naturally derived alkaloids, synthetic alkaloids also exhibit anti-PEDV properties. Three synthetic derivatives of carbazole alkaloids Carbazole Derivatives have demonstrated the ability to inhibit PEDV by obstructing viral adsorption. These compounds are characterized by aromatic and substituted expanded rings, which can be synthetically modified to enhance their anti-PEDV efficacy (43). This finding underscores the potential for developing potent clinical therapeutics against PEDV utilizing alkaloid compounds.

### 2.3 Research on the anti-PEDV activity of polysaccharide compounds

Polysaccharide compounds found in herbal medicine, classified as plant polysaccharides, originate from diverse sources and are fundamental to sustaining normal physiological functions. They exhibit various bioactive properties, including antioxidant, antitumor, antibacterial, antiviral, and immunomodulatory activities (44). Research indicates that polysaccharide compounds found in various herbal medicines, including *Pogostemon cablin* polysaccharides (PCP), *Alpiniae oxyphylla* efructus polysaccharide 3 (AOFP3), polysaccharides from the exocarp of *Ginkgo biloba*, and Astragalus polysaccharide, exhibit anti-PEDV properties.

PCP, primarily composed of polysaccharides, are traditionally employed in the treatment of diarrhea, vomiting, nausea, and fever. Recent studies have successfully isolated four distinct polysaccharides from *Pogostemon cablin*, two of which have demonstrated the ability to inhibit PEDV replication and the other two inhibit PEDV entry and replication, and all four polysaccharides have anti-PEDV activity and antioxidant effects (45). The polysaccharide compound AOFP3, derived from the *Alpinia oxyphylla* fructus, demonstrates multifaceted inhibitory effects on PEDV replication. Firstly, AOFP3 mitigates cellular damage induced by PEDV infection through its antioxidant properties (46). Additionally, AOFP3 competitively impedes the adsorption phase of PEDV by obstructing the binding of the PEDV S protein to porcine aminopeptidase on host cells and by decreasing intracellular cholesterol levels, thereby inhibiting the virus’s invasive phase (47). Furthermore, AOFP3 has been shown to down-regulate the activity of PEDV RNA-dependent RNA polymerase (RdRp) and reduce the levels of heterogeneous nuclear ribonucleoprotein A1 (hnRNPA1) within cells. This action prevents the binding of the 3′ untranslated region (3’UTR) of the PEDV genome to RdRp, thereby disrupting the replication and maturation of PEDV RNA (48). Polysaccharide from *Ginkgo Biloba* Exocarp have demonstrated efficacy in obstructing the adsorption and entry stages of PEDV and possess the capability to directly inactivate the virus. However, this activity is contingent upon specific conditions of time, dosage, and temperature (49). Polysaccharide extracts derived from herbal medicines represent a significant source for the development of potential antiviral agents against PEDV.

### 2.4 Research on the anti-PEDV activity of saponins

Saponins, which are prevalent in medicinal plants, can be categorized into triterpenes and steroidal saponins based on their parent structures. These compounds exhibit a range of pharmacological activities, including antibacterial, anti-inflammatory, hypoglycemic, and immunomodulatory effects (50, 51). Glycyrrhizin (GLY), a saponin analogue isolated from the root of *Glycyrrhiza glabra* L., mitigates the pro-inflammatory response induced by PEDV infection by inhibiting the cytokine activity of high mobility group box 1 (HMGB1), primarily targeting the entry and replication stages of PEDV (52). Subsequent investigations have demonstrated that GLY impedes PEDV infection and diminishes pro-inflammatory cytokine secretion via the HMGB1/toll-like receptor 4 (TLR4)-mitogen-activated protein kinase p38 (MAPK p38) signaling pathway (53). Buddlejasaponin IVb, a naturally occurring triterpenoid saponin isolated from *Pleurotus ostreatus*, has emerged as a promising anti-PEDV compound. It primarily targets the replication and release stages of PEDV and inhibits the activation of the NF-κB signaling pathway, thereby down-regulating the levels of inflammatory factors. The SI values on Vero cell were 12.18 μM. This mechanism effectively alleviates the clinical symptoms and intestinal damage associated with PEDV infection in pigs (54). Additionally, escins, which comprise various saponin mixtures, exhibit cytotoxic effects that are associated with acylations at the C-21 and C-22 positions with angeloyl or tigloyl groups. The study identified that the removal of angeloyl or tigloyl groups at C-21 and C-22, or the modification of glycosidic linkages through hydrolysis, can inhibit PEDV replication at low cytotoxic doses. This suggests potential for these compounds to be developed into highly effective drugs against PEDV (55). A recent study has identified that panax notoginseng saponins (PNS), bioactive extracts derived from *Panax notoginseng*, exhibit anti-PEDV effects in Vero cells. Furthermore, PNS was found to inhibit PEDV during the genome replication stage. mRNA-seq analysis indicated that PNS may exert antiviral effects through a diverse array of molecular pathways and cellular processes (56).

### 2.5 Research on the anti-PEDV activity of other compounds

Ergosterol peroxide (EP) is a steroid derivative that can be extracted from a variety of fungi, yeasts, lichens, or sponges, and exhibits antitumor, pro-apoptotic, anti-inflammatory, antimycobacterial, and antiproliferative properties (57). EP impedes PEDV-induced apoptosis and consequently inhibits PEDV replication by suppressing ROS generation and p53 activation. It acts during the entry, replication, and release phases and directly inactivates the virus (58). Furthermore, prenylated phenolic compounds isolated from the leaves of *Sabia limoniacea* have shown promising anti-PEDV effects, with two compounds exhibiting IC50 values 7.5 ± 0.7 μM and 8.0 ± 2.5 μM, respectively, suggesting their potential as candidates for the development of effective anti-PEDV therapeutics (59). Additionally, a coumarin compound isolated from the roots of *Saposhnikovia divaricata* has been shown to inhibit the synthesis of PEDV structural proteins N and S in a dose-dependent manner (60). Hypericin, a dianthrone compound isolated from *Hypericum perforatum* L., has been shown to inhibit infections by PEDV and TGEV through the disruption of PEDV 3CLpro cleavage activity (61). Similarly, Levistolide A, a significant bioactive constituent of the traditional Chinese medicinal herb *Ligusticum chuanxiong*, exerts its inhibitory effects on PEDV by upregulating the mRNA expression levels of genes associated with endoplasmic reticulum stress and modulating the ROS-mediated endoplasmic reticulum stress response. This mechanism primarily impacts the adsorption or entry phase of PEDV infection (62). Magnolol (MAG), the active compound responsible for the antimicrobial properties of the traditional Chinese medicine *Magnolia officinalis* bark, has been shown to significantly decrease PEDV M protein and mRNA levels, as well as viral titers, in Vero cells at a concentration of 30 μM (63). Hyperoside, derived from *Crataegus*, exhibits anti-PEDV activity by inhibiting the interaction between the PEDV N protein and p53 (64). Additionally, four oleanane triterpenes isolated from the flowers of *Camellia japonica* have been found to inhibit PEDV replication by down-regulating the expression of N, S, and M genes and proteins (65). Sesquiterpenoids extracted from the flowers of *Chrysanthemum indicum* were able to inhibit N and S protein synthesis of PEDV and effectively inhibit viral replication (66). Triacetyl resveratrol (TCRV) is a novel natural resveratrol derivative discovered in recent years, the IC50 values in Vero and IPEC-J2 cells were 42.5 μM and 52.3 μM, and TCRV inhibits PEDV by activating the mitochondria-related caspase pathway to induce early apoptosis (67).

## 3 Progress on the anti-PEDV effects of single herbal extracts

Herbal extracts possess a diverse array of applications within both contemporary medical practices and traditional Chinese medicine. Their uses encompass, but are not limited to, anti-tumor, antibacterial, and antioxidant activities, as well as the treatment of rheumatoid arthritis. Empirical research has demonstrated that water extracts from *Epimedium koreanum*, *Aloe vera*, *Hypericum japonicum*, along with aqueous extracts from *Moringa oleifera* leaves, *Castanea crenata* inner shell, *Glycyrrhiza glabra*., and *Portulaca oleracea* L, exhibit activity against PEDV.

A comprehensive screening of 333 oriental herbs identified KIOM 198, an aqueous extract of *Epimedium koreanum*, as exhibiting the most potent antiviral activity against PEDV in both *in vitro* and *in vivo* animal models, suggesting its potential application in the treatment of PEDV-related diseases in swine (68). Additionally, *Aloe vera* extract (Ae) demonstrated direct inactivation of PEDV, achieving complete inhibition in Vero and IPEC-J2 cells at a concentration of 16 mg/mL, while also reducing viral load and pathological changes in the porcine intestinal tract at a relatively safe dosage of 100 mg/kg body weight (69). The extract of *Hypericum japonicum* (HJ) has been shown to directly inactivate PEDV. At a concentration of 0.25 mg/mL, HJ significantly inhibited the proliferation of PEDV in both Vero and IPI-FX cell lines. Additionally, HJ reduced the viral titer in the intestines of piglets, improved their intestinal microbiota and histopathology, and provided a protective effect against PEDV-induced damage, thereby mitigating diarrhea in piglets (70). The aqueous leaf extract of *Moringa oleifera* has been demonstrated to inhibit apoptosis and the proliferation of PEDV by mitigating PEDV infection-induced ROS and malondialdehyde production. This extract effecetively restores glutathione peroxidase activity, thereby reducing cellular oxidative stress and the expression of inflammatory cytokines, which collectively impede apoptosis and PEDV proliferation, particularly during the virus’s replication phase (71). Additionally, the primary constituent of the aqueous extract of *Portulaca oleracea* L. is polysaccharide, which primarily functions during the adsorption phase of viral infection. It inhibits PEDV replication by suppressing the PEDV-activated NF-κB signaling pathway, leading to a downregulation of cytokine levels (72). The primary constituents of *Castanea crenata* inner shell extract (CISE) are gallic acid and ellagic acid. When extracted with ethanol at a concentration of 30 μg/mL, CISE demonstrated an inhibitory effect on PEDV in Vero cells, achieving up to 90% inhibition. This effect was particularly pronounced during the adsorption and membrane fusion phases of the viral lifecycle. Additionally, CISE exhibited inhibitory activity against other coronaviruses, suggesting its potential as a natural broad-spectrum antiviral agent for the development of anticoronaviral drugs (73). Similarly, *Glycyrrhiza glabra* extract has been shown to inhibit PEDV during the stages of viral attachment, internalization, and replication *in vitro*. Furthermore, licorice extract significantly reduced clinical symptoms, pathological damage, and viral loads in the jejunum and ileum of piglets, thereby increasing the survival rate up to 80% in PEDV infected piglets (74). Recent studies have demonstrated that the aqueous extract of *Portulaca oleracea* (WEPO) exhibits a dose-dependent inhibitory effect on cell pyroptosis induced by PEDV. Furthermore, the individual knockdown of Caspase-1 and Gasdermin D (GSDMD) significantly alters the production of the inflammatory cytokine IL-1β. These findings suggest that WEPO mitigates PEDV infection-induced pyroptosis through the Caspase-1/GSDMD pathway (75).

## 4 Progress on the anti-PEDV effects of compound herbal preparations

The compound herbal preparation, guided by the principles of traditional Chinese medicine, utilizes Chinese herbs as raw materials and undergoes a specific refinement process, resulting in a form of pure natural medicine. A team of researchers designed a MYCI reagent formulated by mixing powdered extracts of *Taraxaci mongolia*, *Viola yedoensis* Makino, *Coptis chinensis* Franch. and *Isatis indigotica* Fortune at a ratio of 1:1:1:2, which was found to mitigate the adverse effects of PEDV infection on the growth performance and intestinal lesions of neonatal pigs (76). Lizhong decoction is a TCM prescription form Treatise on Febrile Diseases, which consists of *Panax ginseng* C. A. Mey., *Zingiber officinale* Roscoe, *Atractylodes macrocephala* Koidz. and *Glycyrrhiza uralensis* Fisch. at a ratio of 1:1:1:1 (Protective effects of Lizhong decoction on ulcerative colitis in mice by suppressing inflammation and ameliorating gut barrier). LZD has been widely used as an effective formula for the spleen deficiency syndrome in Chinese medicine (Lizhong decoction ameliorates pulmonary infection secondary to severe traumatic brain injury in rats by regulating the intestinal physical barrier and immune response). LZD was identified to contain 144 alkaloids and 128 terpenoids, which were effective in inhibiting PEDV replication *in vitro* and *in vivo*. LZD primarily inhibits PEDV during the replication phase of its life cycle, significantly reducing apoptosis in IPEC-J2 and Vero cells. Additionally, LZD decreases viral load in intestinal and visceral tissues, mitigates intestinal pathology, and promotes weight gain in PEDV-infected piglets (77). Investigating the inhibitory effects of these compound herbal preparations on the PEDV and elucidating their mechanisms of action provide valuable clinical insights for the prevention and treatment of PED and other diarrheal diseases.

## 5 Discussion

PED is a highly contagious enteric infectious disease that results in substantial economic losses for the swine industry. Currently, existing vaccines do not effectively control the PEDV epidemic, and there is no specific therapeutic drug available for clinical treatment of PEDV. Researchers both domestically and internationally have utilized traditional herbs and purified natural products, characterized by low toxicity, minimal drug resistance, and abundant availability, as crucial resources for the development of anti-PEDV-specific pharmaceuticals (78). Extensive contemporary studies have demonstrated that herbal monomers, single-flavor herbal extracts, and compound herbal formulations contain a diverse array of natural products exhibiting significant inhibitory effects against PEDV (Table 1).

**Table 1:** Summary of natural herbal products against PEDV.

Compound/Component	Source	Classification	SI(μM)	In vivo or in vitro model	Mechanism of action	Reference
Wogonin	*Scutellaria baicalensis*	Flavonoids	Vero:8 IPEC-J2:8.8	Vero and IPEC-J2 cells	Inhibited PEDV 3CLpro activity	Wang et al. (22)
Xanthohumol	*Humulus lupulus*	Flavonoids	Vero:7.6	Vero-E6 cell	Inhibited PEDV 3CLpro activity	Lin et al. (23)
Quercetin	Rutin	Flavonoids		Vero cell	Interacts with the three active sites of 3CLpro	Li et al. (26)
EGCG	*Camellia sinensis*	Flavonoids		Vero cell	Inhibiting PEDV attachment, entry, replication, and release	Huan et al. (29)
Puerarin	*Pueraria lobata*	Flavonoids		Vero cell/24 7-day-old piglets	Inhibits the activation of the NF-κB signaling pathway and reduced PEDV-induced levels of inflammatory cytokines	Wu et al. (31)
Cinchonine	*Cinchona calisaya* bark	Alkaloids		Vero, ST and LLC-PK1 cells	Inhibition of PEDV by inducing autophagy	Li et al. (30)
Matrine	*Magnolia* bark	Alkaloids		Vero and IPEC-J2 cells	Induce apoptosis to inhibit PEDV replication through MAPK signaling pathway and target S protein	Qiao et al. (37)
Tomatidine	*Lycopersicon esculentum*	Alkaloids	Vero:13.25	Vero, ST, Marc-145, BHK-21 and IPEC-J2 cells	Inhibits PEDV replication by directly obstructing 3CLpro activity	Wang et al. (38)
*Pogostemon cablin* polysaccharides	*Pogostemon cablin*	Polysaccharids		IPEC-J2 cell	Anti-PEDV activity and antioxidant effects	Chen et al. (45)
Glycyrrhizin	*Glycyrrhiza glabra*	Saponins		Vero cell	Inhibiting PEDV infection and diminishes pro-inflammatory cytokine secretion via depended on the HMGB1/TLR4-MAPK p38 pathway	Gao et al. (53)
Hypericin	*Hypericum perforatum* L.	Dianthrone compounds		Vero and ST cells	Disruption of PEDV 3CLpro cleavage activity	Zhang et al. (61)
Oleanane triterpenes	The flowers of *Camellia japonica*	Pentacyclic triterpene compounds		Vero cell	Down-regulating the expression of PEDV N, S, and M genes and proteins	Yang et al. (65)
Sesquiterpenoids	*Chrysanthemum*			Vero cell	Inhibit N and S protein synthesis of PEDV and effectively inhibit viral replication	Liu et al. (66)
Triacetyl resveratrol	Resveratrol			Vero and IPEC-J2 cells	Activating the mitochondria-related caspase pathway to induce early apoptosi	Wang et al. (67)
Hypericum japonicum extract	*Hypericum japonicum*			Vero and IPI-FX cells/14 3-day-old piglets	Inhibit PEDV replication and directly inactivate VIrions	Rao et al. (70)
Lizhong decoction				Vero and IPEC-J2 cells	Inhibit PEDV replication	Chen et al. (77)

While a substantial number of studies have highlighted the significant inhibitory effects of active compounds against PEDV *in vitro*, several notable limitations remain. Currently, the majority of research focuses primarily on the antiviral activity of these compounds, yet often overlooks the comprehensive evaluation of host cytotoxicity. This includes critical indicators such as cell viability and apoptosis, which are essential for understanding the potential side effects of these compounds. Moreover, most studies are confined to *in vitro* experiments, lacking a thorough assessment of compound toxicity in animal models. Important parameters such as acute toxicity and subchronic toxicity remain largely unexplored. Although some active compounds have demonstrated promising antiviral effects in vitro, their efficacy *in vivo* has not been fully validated. This gap severely restricts their potential for further development and application. Therefore, future research must adopt a more comprehensive approach to evaluate the safety and efficacy of these active compounds. This includes determining their safety profile, pharmacokinetics, and therapeutic potential in the context of PEDV prevention, treatment, and control. Only through such rigorous assessments can we facilitate the translation of these promising compounds into practical applications.

Despite the foundational research supporting the development of anti-PEDV-specific drugs from Chinese herbs, numerous challenges remain to be addressed from the stages of development through to application. Firstly, while there exists a diverse array of Chinese herbs, only a limited number have been identified with anti-PEDV properties, necessitating further exploration of herbs with potential anti-PEDV activities. Secondly, it is imperative to investigate the specific components within these Chinese herbs that inhibit PEDV, elucidate their mechanisms of action, and assess their applicability to other viral infections.

**Figure 2:**
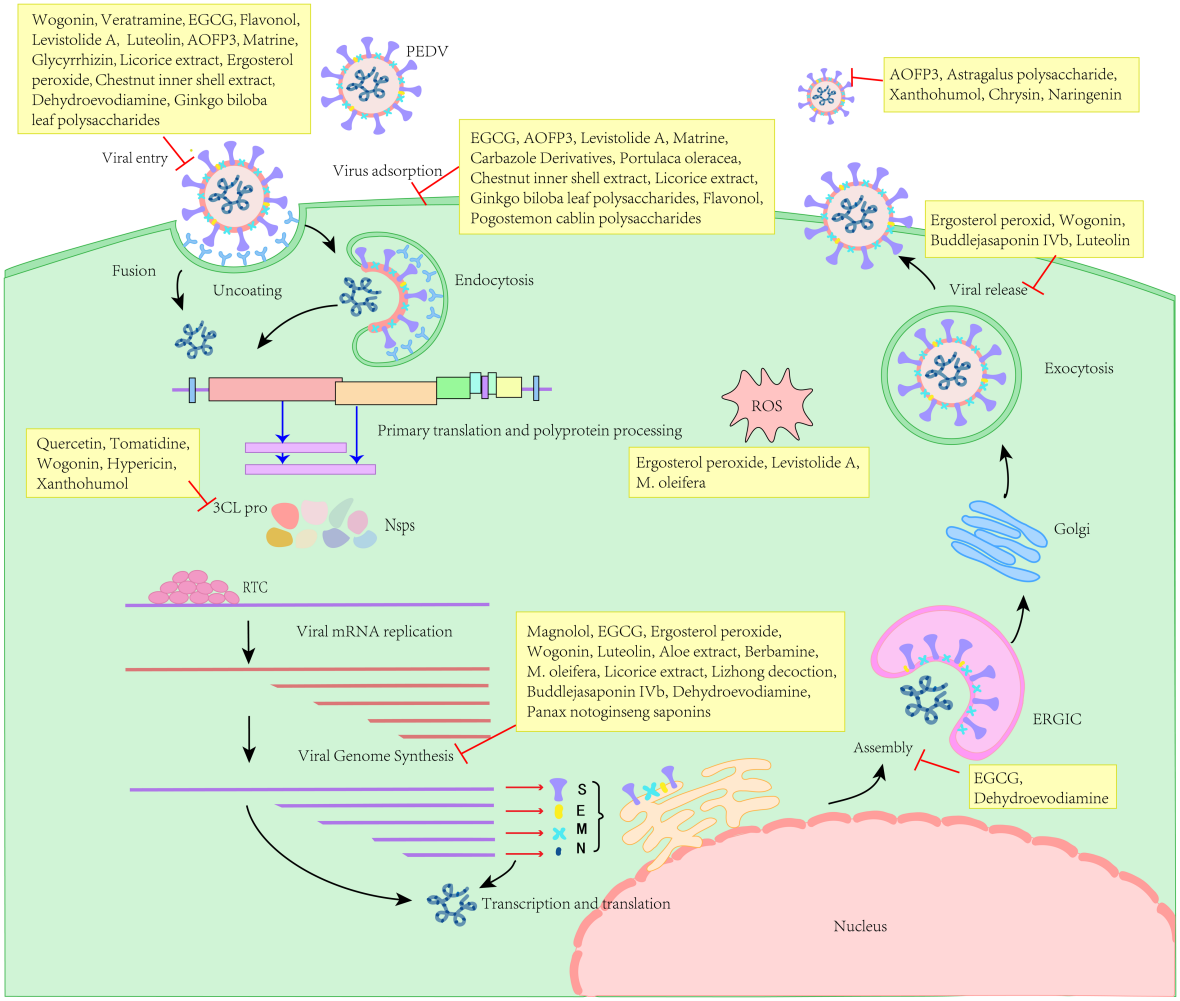
Different natural herbal products target for the life cycle of PEDV.

Furthermore, it is imperative to investigate the optimal clinical utilization of pharmaceuticals derived from herbal medicines, including their potential concurrent use with one another and with vaccines. Although herbal drugs target various stages of the life cycle of PEDV and exhibit distinct mechanisms of action (Figure 2), the precise mechanisms warrant further investigation. Collectively, herbal medicines exhibit significant potential for antiviral applications and offer a theoretical foundation for the development and implementation of antiviral herbal veterinary medicines.

## Glossary

PED: porcine epidemic diarrhea
PEDV: porcine epidemic diarrhea virus
PR: puerarin
EGCG: epigallocatechin-3-gallate
HHT: homoharringtonine
VAM: veratramine
DHED: dehydroevodiamine
VSV: vesicular stomatitis virus
NDV: newcastle disease virus
HSV-1: herpes simplex virus type 1
PRV: pseudorabies virus
HCQ: hydroxychloroquine
PI3K/Akt: phosphatidylinositol 3-kinase/protein kinase B
CEP: cepharanthine
TET: tetrandrine
FAN: fangchinoline
PCP: *pogostemon cablin* polysaccharides
AOFP3: alpiniae oxyphyllaefructus polysaccharide 3
RdRp: RNA-dependent RNA polymerase
hnRNPA1: heterogeneous nuclear ribonucleoprotein A1
HMGB1: high mobility group box 1
TLR4- MAPK p38: toll-like receptor 4-mitogen-activated protein kinase p38
PNS: panax notoginseng saponins
EP: ergosterol peroxide
MAG: magnolol
TCRV: triacetyl resveratrol
Ae: aloe vera extract
HJ: hypericum japonicum
CISE: chestnut inner shell extract
WEPO: aqueous extract of *portulaca oleracea*
GSDMD: gasdermin D
LZD: Lizhong decoction
